# Evaluation of the Antiviral Activity of a Natural Product, Schisandrin B, Against Rhabdovirus Infection in Chinese Rice Field Eels

**DOI:** 10.3390/ijms27021118

**Published:** 2026-01-22

**Authors:** Yisha Liu, Mingyang Xue, Chen Xu, Yong Zhou, Nan Jiang, Yan Meng, Yiqun Li, Zhenyu Huang, Wenzhi Liu, Yuding Fan

**Affiliations:** 1 Yangtze River Fisheries Research Institute, Chinese Academy of Fishery Sciences, Wuhan 430223, China; liuys629@163.com (Y.L.); xmy@yfi.ac.cn (M.X.); xuchen@yfi.ac.cn (C.X.); zhouy@yfi.ac.cn (Y.Z.); jn851027@yfi.ac.cn (N.J.); mengy@yfi.ac.cn (Y.M.); liyq@yfi.ac.cn (Y.L.); hzy@yfi.ac.cn (Z.H.); 2 College of Fisheries and Life Science, Shanghai Ocean University, Shanghai 201306, China

**Keywords:** schisandrin B, rhabdovirus, *Monopterus albus*, antiviral

## Abstract

Chinese rice-field eel rhabdovirus (CrERV), an emerging viral pathogen, causes massive death in rice-field eels (*Monopterus albus*), thus threatening the industry’s development. There is currently no established treatment strategy for CrERV. This study evaluated the anti-CrERV effects of schisandrin B (Sch B) in vitro and in vivo. The results indicated that Sch B at 20 mg/L could inhibit the expression of the CrERV G protein, with a maximum inhibition rate of 69.5%. Additionally, Sch B mitigated the nuclear damage and mitochondrial membrane potential decline induced by CrERV, thereby preserving cellular morphology. A time-of-addition study suggested that Sch B might exert its antiviral effects during the mid-stage of viral replication. In vivo, Sch B exhibited promising preventive and therapeutic effects against CrERV infection in rice-field eels, enhancing their survival rate by 57% and 51%, when added at 0.075% and 0.025%, respectively. Overall, the natural product Sch B was proven to have excellent anti-CrERV activity, with broad prospects for application in aquaculture.

## 1. Introduction

Chinese rice-field eels (*Monopterus albus*) are an important fish in the Chinese freshwater fishery industry and have become a highly sought-after commodity in the fish market in recent years because of their fast growth, high survival rate, high adaptability to net-pen culture conditions, excellent flavor, high nutritional value, and medicinal value [1]. However, the rapid expansion of intensive aquaculture in recent years has led to increased concerns over the frequent occurrence of diseases during the cultivation process. These diseases seriously impair the growth, development, and reproduction of eels, even resulting in their mass mortality, causing significant economic losses [2,3,4,5,6,7]. Chinese rice-field eel rhabdovirus (CrERV), which belongs to the family *Rhabdoviridae*, has been identified as a highly pathogenic viral agent in recent years and has had a significant negative impact on the eel farming industry. The obvious clinical manifestations of CrERV outbreaks encompass cranial enlargement and widespread bodily hemorrhage [7]. CrERV mortality episodes a severe epidemic, causing substantial mortality in adult and juvenile farmed Chinese rice-field eels, resulting immense economic losses [7]. Presently, the absence of efficacious protective measures against CrERV infection in rice-field eels constitutes a substantial health hazard for aquaculture. Therefore, the development of antiviral strategies to address lethal CrERV outbreaks is urgently needed.

In recent years, herbal medicines have gained attention as potential aquaculture additives and antiviral agents due to their broad bioactive components, regulatory effects on biological functions, and environmental safety [8]. Some natural compounds are effective against aquatic viral diseases. For example, emodin inhibits Cyprinid herpesvirus 3 (CyHV-3) replication, reduces CyHV-3-inducedmortality in Koi, and protects against CyHV-3-induced adverse effects. Emodin exerts these effects by improving antioxidant function and alleviating oxidative stress and inflammatory cytokines via the NFE2 like BZIP transcription factor 2 (Nrf2)/Kelch like ECH associated protein 1 (Keap1)–Antioxidant Response Element (ARE) pathway and nuclear factor kappa B (NF-κB) pathway [9]. In addition, in vivo and in vitro studies showed that artemisinin [10] and saikosaponin D [11] are promising drugs for the prevention and treatment of spring viremia of carp virus (SVCV) infections. Schisandrin B (Sch B), a biologically potent biphenyl cyclooctene lignan, is isolated from the fruit of *Schisandra chinensis* [12]. Compared with the other isomers of Sch (Sch A and Sch C), Sch B has a more potent therapeutic potential [13]. Prior research endeavors have established the noteworthy therapeutic potential of Sch B for addressing cancer, cardiovascular diseases, and, furthermore, neurodegenerative disorders [14,15]. It possesses a diverse array of pharmacological effects, notably its antioxidant, anti-inflammatory, and antitumor properties [16,17]. In terms of antiviral activity, in vitro studies have shown that the lignans in *Schisandra. chinensis* have certain antiviral activities against herpes simplex virus (HSV-2), hepatitis B virus, and adenovirus [18,19]. In addition, Schisandra polysaccharides can act as immune activators in aquatic animals. Specifically, the addition of 500 mg/kg of Schisandra chinensis polysaccharide to the basal feed enhanced the innate immune response and resistance to Aeromonas hydrophila in crucian carp (*Carassius auratus*) by enhancing the C3 complement level, superoxide dismutase (SOD) activity, immune cell activity, and antimicrobial enzyme activity [20].

In this investigation, quantitative real-time reverse transcription PCR (RT-qPCR) was employed as a means to assess the antiviral activity of Sch B against CrERV in vitro. By employing cell fluorescence staining, scanning electron microscopy (SEM), and transmission electron microscopy (TEM), in-depth analysis of the effect of Sch B on apoptosis characteristics after CrERV infection of Gibel carp brain (GiCB) cells was carried out. Furthermore, the antiviral efficacy of Sch B against CrERV in vivo was rigorously assessed employing reverse RT-qPCR alongside survival assays. Our results indicated the capability of Sch B to serve as a promising anti-CrERV agent in aquaculture.

## 2. Results and Discussion

### 2.1. Sch B Exhibits Dose-Dependent Antiviral Activity Against CrERV In Vitro

Traditional Chinese herbal medicines represent a promising source for developing novel antiviral agents. In particular, several natural compounds—such as arctigenin, curcumin, and emodin—have recently been reported to exhibit inhibitory effects against various aquatic viruses [21,22,23]. Sch B, a dibenzocyclooctadiene lignan derived from Schisandra fruits, has been widely studied for its anti-inflammatory, antitumor, and antioxidant properties [13,24,25,26]. However, its potential as an antiviral agent, especially against aquatic viruses, remains less explored.

**Figure 1:**
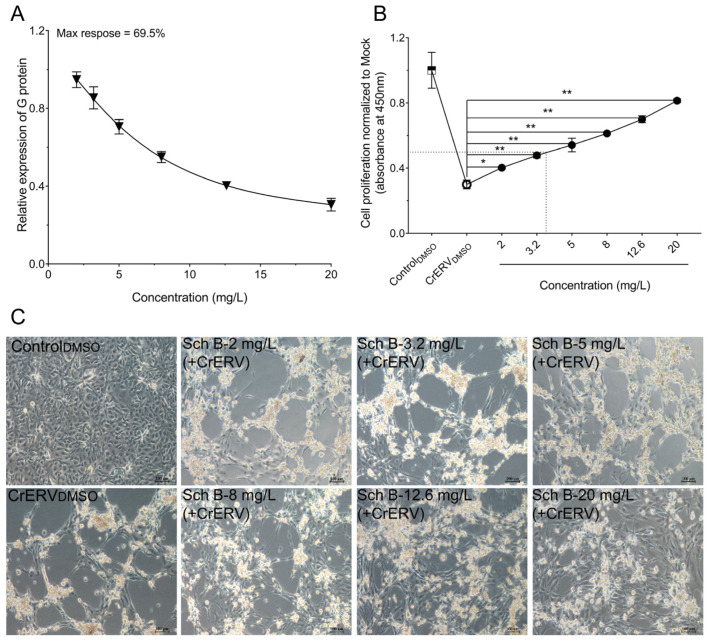
Sch B antiviral activity against CrERV. (**A**) The study was performed using GiCB cells and the antiviral activity of Sch B was determined using a six-point dose–response curve. (**B**) Protective effect of Sch B on the survival rate of GiCB cells. (**C**) In GiCB cells infected with CrERV, Sch B was analyzed for its morphological effects. * *p* < 0.05; ** *p* < 0.01.

In this study, we evaluated the antiviral activity of Sch B against CrERV in GiCB cells using a non-cytotoxic concentration range (2.0–20 mg/L) determined prior to the experiment. Sch B significantly inhibited CrERV infection in a dose-dependent manner, with a maximum inhibition rate of 69.5% (Figure 1A). Furthermore, even at the highest tested concentration of 20 mg/L, Sch B treatment maintained cell viability at 81.37 ± 1.14% (Figure 1B), a level notably higher than that observed in the virus-only control group (where viability was substantially reduced due to infection), thus demonstrating a clear protective effect against virus-induced cell damage. In parallel, Sch B considerably alleviated the virus-induced cytopathic effect (CPE) at 48 h post-infection (Figure 1C). These findings collectively indicate that Sch B not only reduces viral replication but also preserves cellular integrity, underscoring its potential as a candidate for further antiviral development.

### 2.2. Sch B Confers Prophylactic and Post-Infection Efficacy Without Virucidal Activity

**Figure 2:**
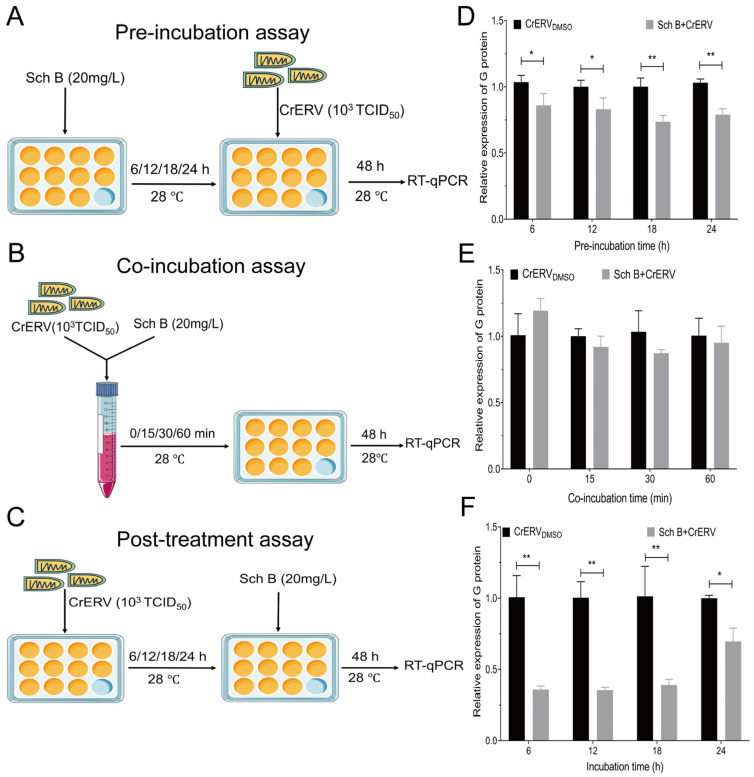
The effect of Sch B on CrERV infection. (**A**–**C**) Schematic of the workflow of pre-incubation, co-incubation, post-incubation assay. (**D**) Pre-incubation. (**E**) Co-incubation of Sch B and CrERV. (**F**) post-incubation. Triplicate experiments yielded mean ± SD values for analysis. * *p* < 0.05; ** *p* < 0.01.

We next evaluated whether Sch B could prevent CrERV infection or directly inactivate viral particles. Pre-incubation with Sch B for 6–24 h prior to viral exposure significantly inhibited CrERV replication (Figure 2D), suggesting a prophylactic potential. However, direct incubation of Sch B with CrERV did not reduce viral infectivity (Figure 2E), indicating that its mechanism is not virucidal. This aligns with reports on other herbal-derived antivirals, such as bavachin and arctigenin, which also act through host-directed mechanisms rather than direct virion disruption [26,27].

### 2.3. Inhibition of CrERV by Schisandrin B Appears to Occur During the Intermediate Phase of Replication

**Figure 3:**
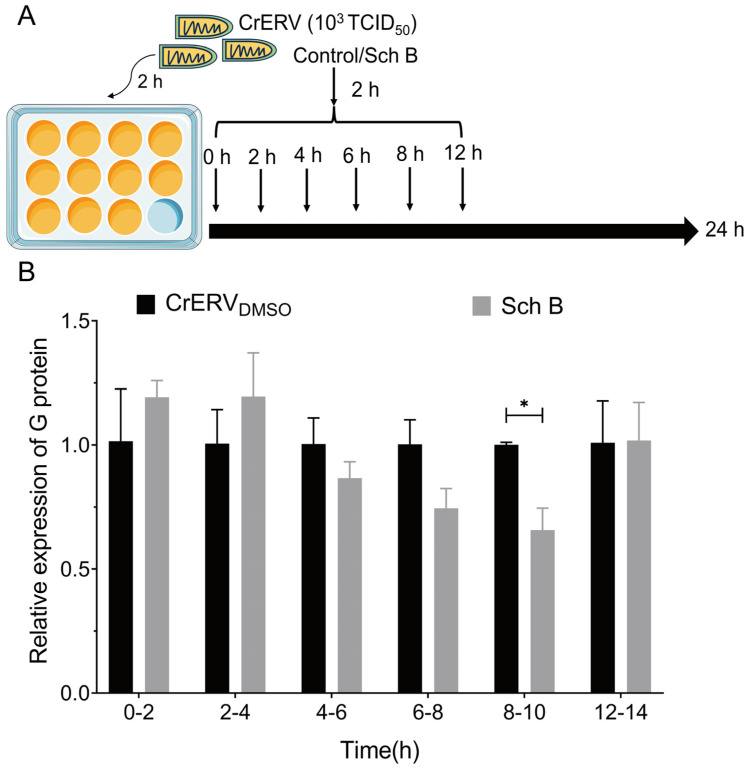
Sch B targets CrERV replication cycle processes. (**A**) Schematic of the workflow of the time-of-addition assay. (**B**) Relative G gene RNA levels were determined by RT-qPCR from the addition assay. Triplicate experiments were conducted and each value represents the mean ± SD. * *p* < 0.05.

To further delineate the mechanism of Sch B, a time-of-addition assay was conducted (Figure 3A). Sch B exerted its strongest inhibitory effect when added at 8–10 h post-infection, reducing G protein expression by 34.23 ± 8.82% (Figure 3B). By contrast, no significant inhibition was observed at other time points. This suggests that schisandrin B may exert its antiviral effect by targeting the intermediate stage of CrERV replication, distinguishing it from arctigenin and bavachin, which primarily act during the early viral entry or initial replication phases [26,27]. The specific molecular mechanism underlying this inference—drawn from the time-of-addition assay—requires further validation through experiments such as Western blot analysis, which will be employed in future studies to examine the expression profiles of key viral proteins during the intermediate phase of replication. It is noteworthy that the finding that Sch B acts during the intermediate stage of CrERV replication shares certain commonalities with the reported mode of action in which Sch B impairs the early phase of HIV-1 replication by inhibiting reverse transcriptase activity [28]. This observation further suggests that Sch B may possess a broad-spectrum antiviral mechanism across different virus families, thereby providing a rationale for its development as a promising lead compound with pan-antiviral potential.

### 2.4. Sch B Attenuates CrERV-Induced Apoptosis via Mitochondrial Protection

**Figure 4:**
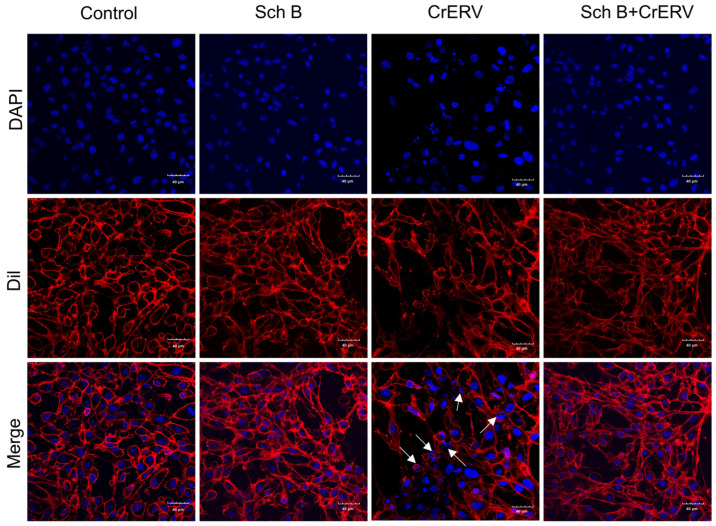
Sch B reduces the number of apoptotic vesicles in GiCB cells. Blue is utilized to signify the nucleus, whereas red designates the cellular membrane, and white arrows are employed to point towards apoptotic bodies. DAPI, 4′,6-diamidino-2-phenylindole; Dil, 1,1′-Dioctadecyl-3,3,3′,3′-Tetramethylindocarbocyanine Perchlorate.

**Figure 5:**
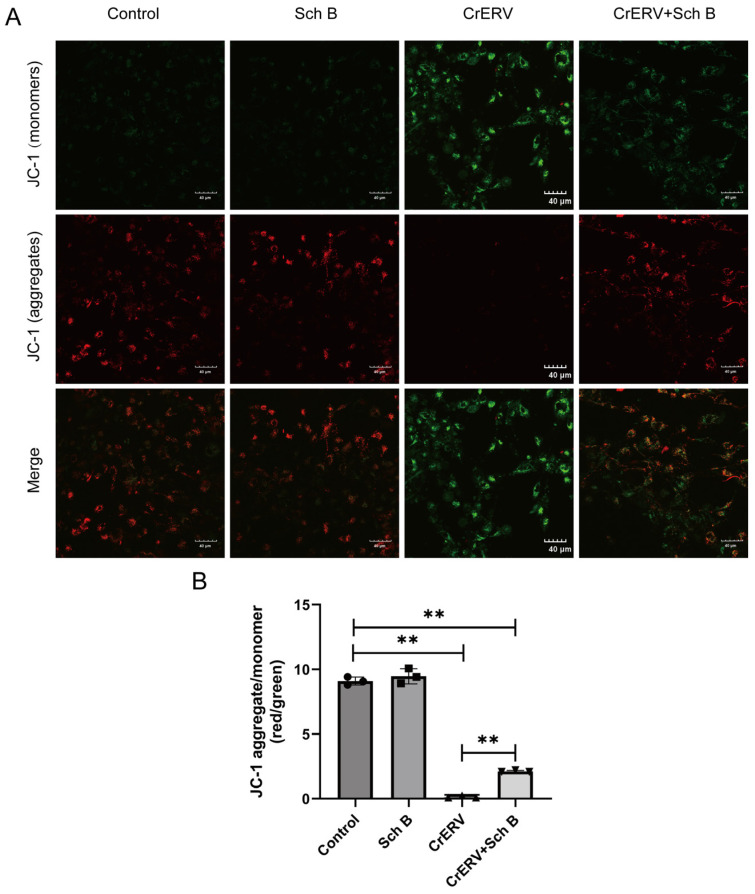
Sch B inhibits CrERV-induced changes in MMP. (**A**) JC-1 fluorescence (confocal microscopy). Red fluorescence (J-aggregates) indicates high mitochondrial membrane potential (∆Ψm); green fluorescence (monomers) indicates low ∆Ψm (depolarization). (**B**) Quantitative analysis of mitochondrial membrane potential, expressed as the JC-1 red/green fluorescence intensity ratio (mean ± SD; >30 cells analyzed per group using ImageJ 1.54g).Data are mean ± SD of three independent experiments. ** *p* < 0.01.

Apoptosis is a common strategy employed by viruses to facilitate dissemination and evade host immune responses [10,26]. Rhabdoviruses can trigger apoptosis through mitochondrial permeabilization, ER stress, or immunopathological signaling [24,25,26]. In this study, we found that CrERV infection induced classical morphological hallmarks of apoptosis in GiCB cells, including nuclear fragmentation and loss of membrane integrity (Figure 4), concomitant with a significant reduction in MMP (Figure 5). Quantitative analysis further revealed that infection significantly decreased the red-to-green fluorescence intensity ratio of JC-1 staining. Treatment with Sch B effectively ameliorated this loss, resulting in a fluorescence ratio that was significantly higher than that of the virus-infected group, yet still significantly lower than that of the normal control group (Figure 5B). Given that the collapse of MMP is a pivotal early event in the apoptotic cascade, the quantitative JC-1 data presented here provide crucial functional evidence for the anti-apoptotic effect of Sch B. Previous studies have also indicated that Sch B can exert its anti-apoptotic activity by modulating key proteins in the mitochondrial apoptotic pathway, such as Bax/Bcl-2, and by influencing mitochondrial function, including the stabilization of MMP, to inhibit apoptosis [29,30]. Collectively, these findings demonstrate that Sch B treatment significantly alleviates virus-induced apoptotic phenotypes, helping to preserve nuclear integrity and attenuate MMP collapse.

In conclusion, our results demonstrate that Sch B mitigates virus-induced apoptosis by ameliorating the infection-mediated loss of mitochondrial membrane potential (Figure 5B). Its partial restoration of MMP is consistent with reports in other pathological contexts and may be attributable to its documented antioxidant activity [16,31,32]. By mitigating MMP collapse and the ensuing activation of the caspase pathway, Sch B helps sustain cell viability, thereby restricting viral replication and spread.

### 2.5. Sch B Preserves Cellular Ultrastructure Integrity

**Figure 6:**
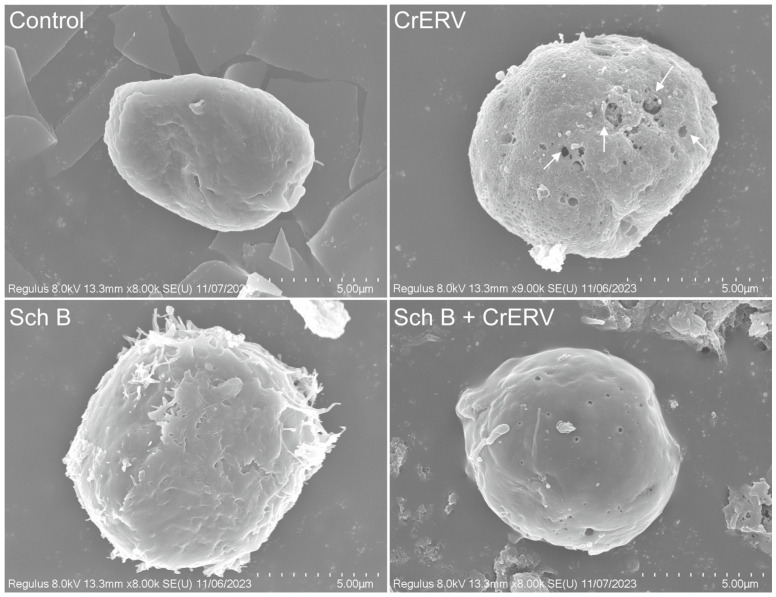
Scanning electron microscopy of the morphoprotective effect of Sch B on GiCB infected with CrERV. White arrows indicate the pores formed due to viral infection.

The scanning electron microscopy results (Figure 6) showed that the cell morphology of the control group remained intact and the surface was relatively smooth. By contrast, 48 h of CrERV infection significantly altered cell morphology, resulting in a rough, uneven surface with varying pore formation (Figure 6, white arrows). Concurrently, the cells subjected to Sch B treatment retained an optimal morphology and structural integrity, demonstrating no discernible variations when juxtaposed against the control cohort.

**Figure 7:**
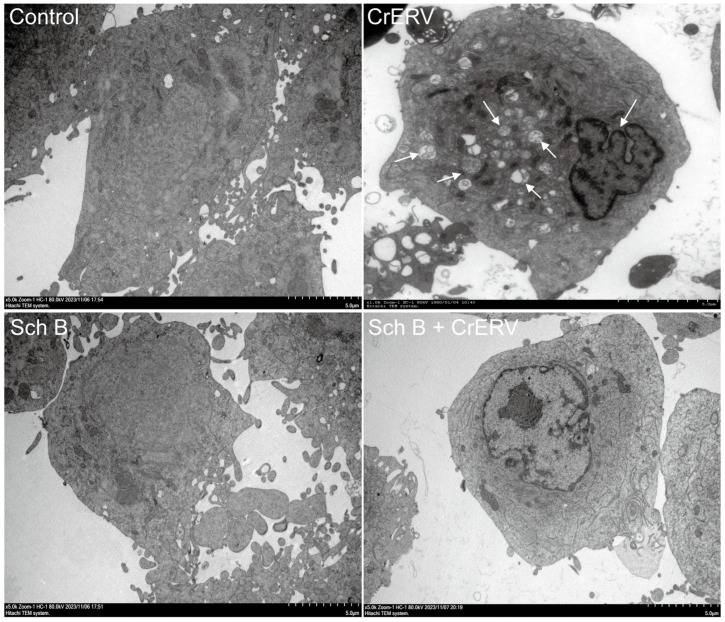
Transmission electron microscopy observations of the morphoprotective effect of Sch B on GiCB cells infected with CrERV. White arrows indicate chromatin condensation and vacuolization in virus-infected cells.

Figure 7 displays the results of transmission electron microscopy (TEM) observation on the internal ultrastructure of cells infected with CrERV and the protective effects of the drug on these cells. As shown by the arrows, CrERV-infected cells exhibited chromatin condensation, nuclear membrane separation, and vacuolization. However, no notable variations were detected between the cells subjected to Sch B treatment and those in the control group. Additionally, the internal structures of the nucleus, mitochondria, and other organelles remained intact after Sch B treatment. Notably, the nuclei, mitochondria, and other organelles remained intact. However, it should be noted that the SEM/TEM analyses in this study primarily provide qualitative morphological evidence. While these images offer visual demonstration of the protective effects of Sch B on cellular ultrastructure, future studies could establish semi-quantitative scoring systems to allow for more objective assessment of the extent of damage.

The protective effect of Sch B on cellular ultrastructure observed in this study shares mechanistic similarities with findings from other pathological models. For instance, research has confirmed that Sch B can significantly improve mitochondrial membrane potential and enhance mitochondrial function by upregulating key proteins such as Sirt3, thereby inhibiting apoptosis [29]. Concurrently, Sch B has also been demonstrated to alleviate endoplasmic reticulum (ER) stress, a cellular pathological process closely associated with viral infection [33]. Therefore, we speculate that the anti-CrERV efficacy of Sch B may stem from a multi-target cytoprotective strategy: while directly inhibiting viral replication, it maintains the overall structural and functional homeostasis of cells by enhancing the cellular antioxidant status (e.g., elevating mitochondrial glutathione levels) and mitigating stress damage to organelles such as the ER [29,33,34]. This protective effect on host cells creates an unfavorable intracellular environment for viral replication and dissemination.

### 2.6. Dietary Sch B Enhances Immune Responses in Rice-Field Eels

**Figure 8:**
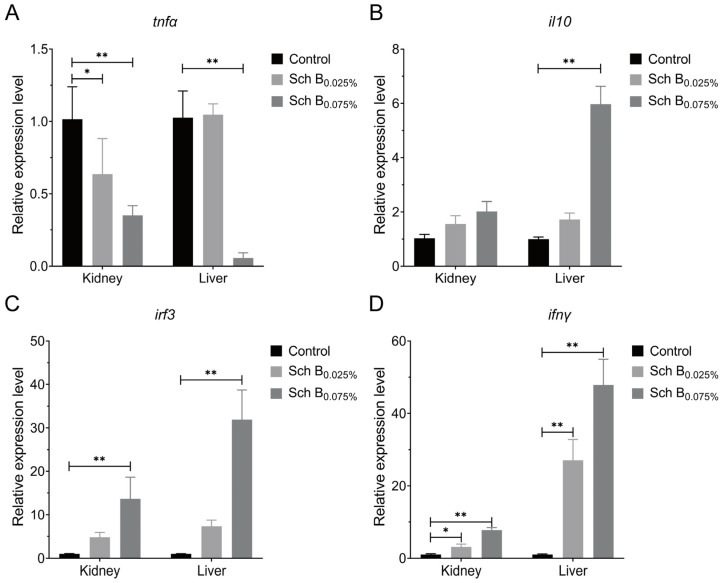
Changes in Sch B immunity genes after 7 days of feeding. (**A**) Alterations in the relative expression levels of *tnfα*. (**B**) Alterations in the relative expression of *il10*. (**C**) Alterations in the relative expression of *irf3*. (**D**) Alterations in the relative expression of *ifnγ*. Mean ± SD (*n* = 3), * *p* < 0.05; ** *p* < 0.01.

Chinese herbal medicines are commonly used as immunopotentiators in aquaculture. Adding Chinese herbal monomers to feed or by absorption methods for fish can effectively improve the immunity of fish and prevent viral infection. Ingesting Astragalus polysaccharides in the diet enhances the serum antioxidant potential and immunological indices of *Larimichthys crocea*, thereby safeguarding it against Vibrio alginolyticus infection [35]. Diets supplemented with 0.1%, 0.5%, and 1% arctigenin daily significantly enhanced the survival of MSRV-infected largemouth bass [21]. To assess the immunomodulatory potential of Sch B in vivo, rice-field eels were fed a diet supplemented with 0.025% or 0.075% Sch B for 7 days. RT-qPCR analysis revealed that 0.075% Sch B significantly downregulated tnfα in both liver and kidney, while upregulating il10, irf3, and ifnγ (Figure 8). The low-dose group (0.025%) also showed reduced tnfα in the kidney and elevated ifnγ in both tissues. These results indicate that Sch B enhances anti-inflammatory and antiviral immune pathways, consistent with previous reports on herbal immunopotentiators in aquaculture [36,37].

### 2.7. Effect of Sch B on In Vivo Survival and Viral Loads

To systematically evaluate the antiviral potential of Sch B in vivo, this study designed both prophylactic and therapeutic administration regimens as a preliminary efficacy assessment. It should be noted that the selected concentrations in the prophylactic model (0.025% and 0.075%) were based on observations from preliminary experiments, aiming to verify observable protective activity and preliminarily explore the effective dose range to inform future, more systematic gradient dose studies.

**Figure 9:**
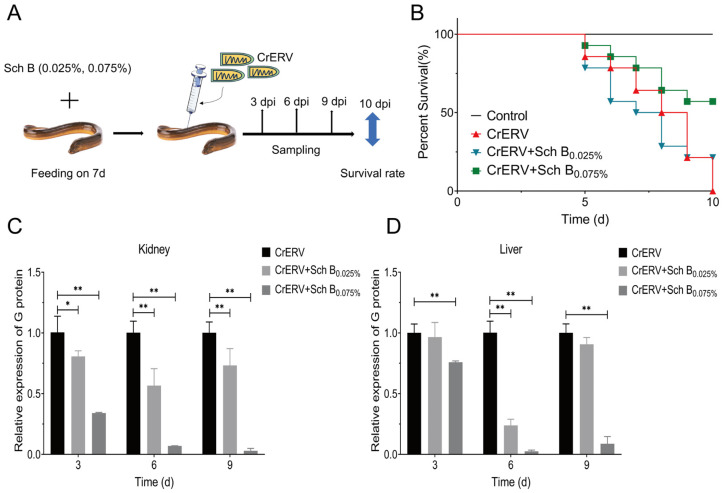
Preventive effect of Sch B on CrERV-infected rice-field eels. (**A**) Schematic of the workflow of the in vivo assay of Sch B antiviral activity. (**B**) Survival curves over 10 days. (**C**,**D**) Viral load analyses of rice-field eel livers and kidneys at 3, 6, and 9 dpi. Mean ± SD (*n* = 3), * *p* < 0.05; ** *p* < 0.01.

**Figure 10:**
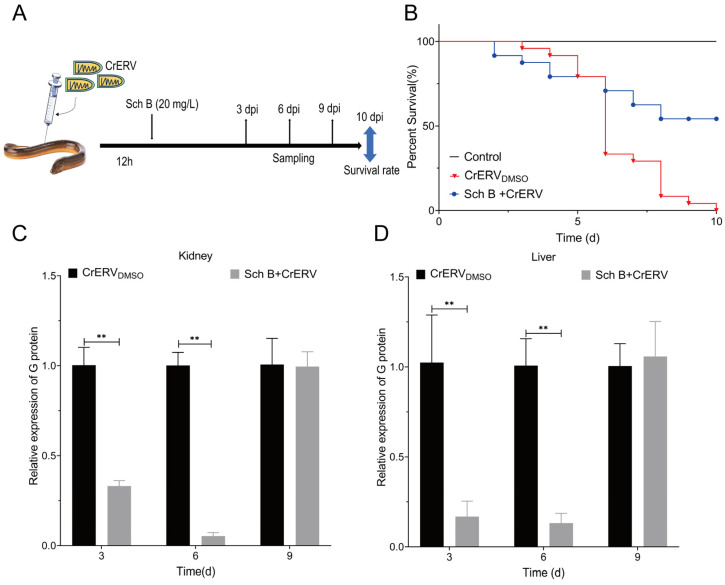
Protective effect of Sch B on CrERV-infected rice-field eels. (**A**) Schematic of the workflow of the in vivo assay of Sch B antiviral activity. (**B**) Survival curves over 10 days. (**C**,**D**) Viral load analyses of rice-field eel livers and kidneys at 3, 6, and 9 dpi. Mean ± SD (*n* = 3), ** *p* < 0.01.

Comprehensive assessment was performed through monitoring survival rates and quantifying tissue viral loads. In the prophylactic experiment, rice-field eels fed with Sch B-supplemented diets exhibited significantly higher survival rates following CrERV challenge. Specifically, the 0.075% Sch B group achieved a survival rate of 57%, while the 0.025% Sch B group showed a 21% increase in survival rate compared to the virus-infected control group (Figure 9B). Furthermore, viral loads in both liver and kidney tissues were significantly reduced in all Sch B-treated groups (Figure 9C,D). In the therapeutic model, oral administration of Sch B at 12 h post-infection resulted in a relative survival rate of 54% (Figure 10B) and significantly reduced tissue viral loads at early time points post-infection (Figure 10C,D). Collectively, these in vivo results demonstrate that Sch B possesses both prophylactic and therapeutic efficacy against CrERV infection, highlighting its potential as a feed additive or oral antiviral agent.

This study demonstrates that Sch B possesses both prophylactic and therapeutic efficacy against CrERV infection in rice-field eels, highlighting its potential as a feed additive or oral antiviral agent. It should be noted that the present work primarily provides a foundational evaluation of its acute effects. To fully assess its practical applicability, several aspects warrant further investigation. First, the long-term safety and potential risk of resistance development upon sustained use require evaluation. Second, its ecological impact on non-target organisms (e.g., phytoplankton and zooplankton) must be assessed. Notably, studies have shown that aqueous extracts of Schisandra chinensis exhibit acute toxicity to aquatic crustaceans such as Daphnia magna (48 h EC_50_ as low as 0.0152 mg/L) [38], underscoring the need for rigorous environmental risk assessment prior to large-scale application. Finally, the precise molecular mechanism of action, as well as its comparative advantage relative to other reported organic agents against fish rhabdoviruses [39], remains to be elucidated.

## 3. Materials and Methods

### 3.1. Cell, Virus, Fish, and Medicine

The CrERV-sensitive GiCB cell line used in this study was established in our laboratory [40]. CrERV was propagated in GiCB cells until manifestation of a cytopathic effect (CPE). Subsequently, the cells from the culture were collected and stored at −80 °C for future experimentation. Viral titer was quantified via TCID_50_ (50% tissue culture infectious dose) analysis, employing the Reed-Muench methodology [11].

Prior to infection, 10 randomly selected Chinese rice-field eels (11–16 cm) from 300 healthy specimens obtained from a farm with no recorded history of CrERV outbreaks were screened for CrERV via triplicate RT-PCR targeting the viral nucleoprotein gene [7]. This RT-PCR assay detects viral RNA, thereby confirming the absence of ongoing viral replication in the selected individuals at the time of screening. Negative controls ensured assay validity. Furthermore, to minimize the risk of pre-existing immunity, eels were acclimatized under strict biosecure conditions. Confirmed CrERV-negative eels were acclimatized for 7 days in controlled systems (25 ± 0.5 °C; 6.5 ± 0.2 mg/L DO) with pathogen-free feed and daily 30% water renewal. Aladdin Chemistry (China) provided Sch B. Sch B was prepared in dimethyl sulfoxide (DMSO) to create a stock solution with a concentration of 50 mg/mL.

### 3.2. Cell Viability Assay

To establish a safe concentration range for subsequent antiviral experiments, the cytotoxicity of Sch B on GiCB cells was first evaluated. According to the method described in [41], cells were exposed to a series of gradient concentrations of Sch B. Cell viability was measured using the Cell Counting Kit-8 (CCK-8) assay, and morphological observation under a microscope (cytopathic effect, CPE) was performed for comprehensive assessment. Preliminary screening identified 20 mg/L as the maximum non-cytotoxic concentration, at which cell viability remained >90% with no obvious drug-related toxicity observed.

Based on this safe concentration upper limit, a finer concentration gradient (20, 12.6, 8, 5, 3.2, and 2 mg/L) was further established using a geometric dilution method to verify the protective effect of Sch B on virus-infected cells within the safe concentration range. Briefly, GiCB cells were incubated with the respective concentrations of Sch B (prepared by diluting the stock solution in serum-free M199 medium, Hyclone, Logan, UT, USA) for 48 h. Subsequently, cell viability was detected using the Cell Counting Kit-8 (CCK-8, Beyotime, Nantong, China), and absorbance was measured with a multifunctional microplate reader (BioTek, Winooski, VT, USA). Cell viability calculation:

Cell viability (%) = [(OD_treated_ − OD_blank control_)/(OD_untreated control_ − OD_blank control_)] × 100%

### 3.3. Antiviral Activity of Schisandrin B In Vitro

Cells seeded in 12-well plates were treated with 10^3^ TCID_50_/mL of CrERV for 2 h, and then, Sch B was added at the maximum safe concentration (20 mg/L), based on the results of the toxicity test in Section 2.2. For the vehicle only control, DMSO (*v*/*v* = 0.12%) was employed. After 48 h of culture, the CPE was observed and photographed under a microscope. Cells were then collected for RT-qPCR analysis to detect the expression of the CrERV glycoprotein gene and to assess its antiviral activity.

After a specific operation described in Section 2.2, the CCK-8 method was employed to ascertain the impact of Sch B on the growth activity of cells following CrERV infection.

### 3.4. Co-Incubation, Pre-Incubation, and Post-Treatment Assays

During the pre-incubation assay, cells were incubated with Sch B (20 mg/L) for 6, 12, 18, and 24 h at 28 °C, after which Sch B was removed and CrERV was immediately added. In the co-incubation assay, a mixed suspension of CrERV (10^3^ TCID_50_) and Sch B (20 mg/L) was prepared and incubated with the cells as a monolayer in a 12-well plate at 28 °C for 0, 15, 30, and 60 min. After co-incubation, the solution was changed to cell maintenance solution. In the post-treatment assay, CrERV virus solution (10^3^ TCID_50_) was added to 12-well plates containing cell monolayers. Following a 2 h incubation at 28 °C, the virus solution was removed, and Sch B (20 mg/L) was applied for 6, 12 and 18 h. All samples were obtained 48 h after CrERV infection for quantification of glycoprotein gene expression using RT-qPCR.

### 3.5. Time-of-Addition Study

The time-of-addition assay followed a previously described protocol [42]. Cells infected with CrERV were subjected to treatment with Sch B (20 mg/L) for a duration of 2 h at distinct time points during various stages of the CrERV replication cycle, specifically at 2, 4, 6, 8, and 12 h post-infection. Cells were collected at 24 h after infection and analyzed using RT-qPCR.

### 3.6. Cell Nuclear Damage Assay and Ultrastructural Observation

The cells were exposed to 10^3^ TCID_50_/mL of CrERV for 2 h, followed by co-incubation with 20 mg/L of Sch B at 28 °C for 48 h. Following this, fluorescence observation was conducted to assess nuclear damage, in accordance with previous studies [43]. Fluorescence was observed under confocal microscope (Olympus, Tokyo, Japan).

For ultrastructural observations, cells infected with CrERV and then exposed to Sch B (20 mg/L) were harvested 48 h post-infection and underwent overnight fixation at 4 °C within a 2.5% glutaraldehyde solution. The cell samples were meticulously processed in accordance with the methodology elaborated by Boudjemaa et al. [44], and subsequently observed using SEM (Hitachi, Tokyo, Japan) and TEM (Hitachi, Tokyo, Japan).

### 3.7. Mitochondrial Membrane Potential Assay

The cells underwent infection with CrERV at a titer of 10^3^ TCID_50_/mL and were subsequently subjected to treatment with 20 mg/L of Sch B. After 48 h, the MMP was quantitatively assessed using a highly specific JC-1 assay kit (Beyotime, Nantong, China). Fluorescence changes reflecting the levels of JC-1 aggregates and monomers were visualized through the employment of a confocal microscope. For quantitative analysis, at least three random fields per sample were imaged. The mean fluorescence intensity of the red and green channels for individual cells was measured using the Region of Interest (ROI) tool in ImageJ 1.54g (Fiji distribution). The background intensity from a cell-free area was subtracted for each channel before calculating the red-to-green fluorescence intensity ratio. A minimum of 30 cells per condition per experiment were analyzed.

### 3.8. Measurement of Immune-Related Gene Expression In Vivo

Twelve eels in each group were sequentially fed a basal diet containing 0.025% and 0.075% of Sch B for 7 days, while the control group was fed the basal diet. On the 7th day of feeding, the extent of expression of immune-related genes, including *tnfa* (encoding tumor necrosis factor alpha), *il10* (encoding interleukin 10), *irf3* (encoding interferon regulatory factor 3), and *ifnγ* (encoding interferon gamma) were measured in the liver and head kidney of eels using RT-qPCR.

### 3.9. Antiviral Activity of Sch B In Vivo

To explore the therapeutic and prophylactic effects of Sch B on CrERV infection in Chinese rice-field eels, two distinct treatment strategies—prophylactic and therapeutic—were administered to healthy rice-field eels.

The preventive experiment was conducted through dietary administration by incorporating Sch B into formulated feeds. Rice field eels in the control group were fed a standard basal diet, while the experimental group was divided into two subgroups (*n* = 30 each) receiving the basal diet supplemented with Sch B at concentrations of 0.025% and 0.075%, which were selected to represent a gradient spanning typical pharmacological exposure levels reported in aquatic species [21]. These concentrations were ultimately determined based on observations of fish growth status and tolerance in our preliminary experiments. These concentrations were confirmed through preliminary screening to ensure compatibility with fish physiological tolerance thresholds, showing no significant adverse effects on survival rate, feeding behavior, or hepatic histoarchitecture. After 7 days of feeding, each eel was challenged via intraperitoneal injection with 50 μL of CrERV suspension, having a titer of 10^6.5^ TCID_50_/mL. This delivered a total viral dose of 10^5.0^ TCID_50_ per fish.

An infected group and a treated group of 30 rice-field eels were set up in the treatment trial. Eels in both the treatment and virus-infected groups received an intraperitoneal injection of 50 μL of CrERV suspension per fish (titer: 10^6.5^ TCID_50_/mL), equating to a total challenge dose of 10^5.0^ TCID_50_ per eel. At 12 h after injection of the virus-infected group, 50 μL of a solution containing 20 mg/L Sch B prepared with cell maintenance fluid was orally administered to the treatment group. This administration concentration was selected based on its effective and safe profile observed in preliminary cell and animal tests.

The survival rate of eels was observed and recorded every 24 h for 10 days. Four eel samples were collected for viral load determination at time points on days 3, 6 and 9 after virus infection.

### 3.10. Total RNA Extraction and Gene Quantification

Total RNA was isolated using the Total RNA Extraction Kit (Yeasen, Shanghai, China) and subsequently reverse-transcribed into cDNA employing the Hifair^®^ III 1st Strand cDNA Synthesis SuperMix for qPCR (+gDNA Digester plus) (Yeasen, Shanghai, China). The primers targeting the CrERV glycoprotein (G) gene were designed and synthesized based on the published sequence in reference [7]. All cDNAs were stored at −20 °C until use. Table 1 shows the primers used for RT-qPCR analysis [7,45,46,47,48]. The Rotor-Gene Q instrument (QIAGEN, Dusseldorf, Germany) was utilized to conduct RT-qPCR, incorporating the Hieff UNICON^®^ Universal Blue qPCR SYBR Green Master Mix (Yeasen, Shanghai, China). Amplification was carried out according to the following program: 95 °C for 10 min; followed by 40 cycles of 95 °C for 15 s, 60 °C for 15 s, and 72 °C for 30 s. Each reaction was run in triplicate, and melt-curve analysis was included to verify amplification specificity.

### 3.11. Statistical Analysis

Data are presented as the mean ± standard deviation (SD) from at least three independent experiments. GraphPad Prism 9 (GraphPad Software Inc., La Jolla, CA, USA) was used for all statistical analyses and graph generation. For comparisons between two groups, statistical significance was assessed using the unpaired two-tailed Student’s *t*-test. Comparisons among multiple groups were performed by one-way analysis of variance (ANOVA), followed by Tukey’s post hoc test for pairwise comparisons. Relative mRNA expression levels were quantified using the 2^−ΔΔCt^ method [49]. Differences were considered statistically significant at * *p* < 0.05 and highly significant at ** *p* < 0.01.

## 4. Conclusions

In summary, Sch B showed effective antiviral activity against CrERV in vivo and in vitro. Sch B mainly acts on the middle stage of virus replication, reduces CrERV-induced nuclear damage and mitochondrial membrane potential decline, protects cell morphology, and inhibits CrERV infection. In addition, adding an appropriate amount of Sch B to the diet can regulate the expression of immune-related genes in Chinese rice-field eel to improve its viral resistance. The outcomes of this study indicate that Sch B represents a promising anti-CrERV therapeutic agent with potential applications in the Chinese rice-field eel aquaculture industry.

## Figures and Tables

**Table 1:** Primers employed for RT-qPCR.

Gene		Primer Sequences (From 5′ to 3′)
CrERV-*g*	Forward	GCAAGCTTCCAAGGCCACTT
	Reverse	GCAGACAGCACGTCCGATTC
Gi-*β-actin*	Forward	GATGATGAAATTGCCGCACTG
	Reverse	ACCGACCATGACGCCCTGATGT
Mal-*EF1α*	Forward	ATCCGTCGTGGATATGTGGC
	Reverse	AGCACTGGGGCATAACCTTC
Mal-*tnfα*	Forward	TCCGCTTCCTGGAGTTTGAT
	Reverse	CCAGCAAAGCCTGAGACATC
Mal-*il10*	Forward	TTTGCCTGCCAAGTTATGAG
	Reverse	CATTTGGTGACATCGCTCTT
Mal-*irf3*	Forward	TAAGGCCTGGGCAGAGGTAA
	Reverse	CCAGCGAAACACTTTGTGGG
Mal-*ifnγ*	Forward	ATCCGTCGTGGATATGTGGC
	Reverse	AGCACTGGGGCATAACCTTC

## Data Availability

The original contributions presented in this study are included in the article. Further inquiries can be directed to the corresponding author.
